# Conquering viral drug resistance: Structural and mechanistic paradigms for antiresistance drug design

**DOI:** 10.1016/j.pscia.2025.100094

**Published:** 2025-09-22

**Authors:** Mei Wang, Haiyong Jia, Xinyong Liu, Peng Zhan

**Affiliations:** a State Key Laboratory of Discovery and Utilization of Functional Components in Traditional Chinese Medicine, Department of Medicinal Chemistry, School of Pharmaceutical Sciences, Cheeloo College of Medicine, Shandong University, Jinan, Shandong, China; b School of Pharmacy, Shandong Second Medical University, Weifang, 261053, Shandong, China

**Keywords:** Drug resistance, Drug design, Structural virology, Molecular dynamics, Protein oligomerization, AI-Driven drug discovery

## Abstract

Viral drug resistance remains a critical challenge in antiviral therapy. This perspective highlights five studies on severe acute respiratory syndrome coronavirus 2 (SARS-CoV-2), human immunodeficiency virus type 1 (HIV-1), monkeypox virus (MPXV), influenza A virus (IAV), and Hepatitis B virus (HBV), revealing novel resistance mechanisms and innovative strategies. For SARS-CoV-2, GC376's flexible benzyl group overcomes nirmatrelvir resistance. HIV-1's non-nucleoside reverse transcriptase inhibitors (NNRTIs) 5i3 adapts to resistant mutants via a quinazoline scaffold, while MPXV's tecovirimat acts as a “molecular glue” stabilizing F13 dimers. Expanding these paradigms, we present groundbreaking insights: An indazole-based IAV inhibitor (compound 24) disrupts the conserved PA-PB1 heterodimer, showing sub-micromolar potency against resistant strains. For HBV, a hydrophobic tagging degrader (HyT-S7) induces HBc degradation, bypassing resistance mutations impairing traditional capsid modulators. Key strategies include dynamic flexibility, multivalent interactions, and oligomerization control, integrated with AI-driven design and real-time surveillance. This perspective bridges structural insights with translational applications, offering a roadmap for next-generation, mutation-resilient antivirals.

**Table:** List of abbreviations

SARS-CoV-2	Severe Acute Respiratory Syndrome Coronavirus 2
HIV-1	Human Immunodeficiency Virus Type 1
MPXV	Monkeypox Virus
IAV	Influenza A Virus
HBV	Hepatitis B Virus
NNRTIs	Non-Nucleoside Reverse Transcriptase Inhibitors
NIR	Nirmatrelvir
BLI	Bio-Layer Interferometry
WT	Wild-Type
MD	Molecular Dynamics
RMSF	Root-Mean-Square Fluctuation
RPV	Rilpivirine
ETR	Etravirine
NNIBP	NNRTI Binding Pocket
NRTIs	Nucleoside RT Inhibitors
TPV	Tecovirimat
RdRp	RNA-Dependent RNA Polymerase
cccDNA	Covalently Closed Circular DNA
HyT	Hydrophobic Tagging
AI	Artificial Intelligence
SAR	Structure-Activity Relationships
TDM	Therapeutic Drug Monitoring

The Elusive Challenge of Viral Adaptation to Therapeutic Pressures Viral drug resistance represents one of the most formidable challenges in modern medicine [[1], [2], [3]], undermining the efficacy of antiviral therapies against a spectrum of pathogens, from RNA viruses like severe acute respiratory syndrome coronavirus 2 (SARS-CoV-2) [4], human immunodeficiency virus type 1 (HIV-1) [5] and influenza A virus (IAV) [6] to DNA viruses such as monkeypox virus (MPXV) [7] and Hepatitis B virus (HBV) [8]. The ability of viruses to rapidly evolve through genetic mutations—whether single-point alterations or complex multi-mutation combinations—enables them to evade drug binding, disrupt therapeutic mechanisms, and ultimately compromise clinical outcomes. For instance, the emergence of SARS-CoV-2 variants resistant to oral protease inhibitors like nirmatrelvir (NIR), the proliferation of HIV-1 strains with cross-resistance to non-nucleoside reverse transcriptase inhibitors (NNRTIs), and the rise of tecovirimat-resistant MPXV isolates underscore this challenge. Similarly, the development of IAV strains resistant to neuraminidase inhibitors (e.g., oseltamivir H275Y) and PA endonuclease inhibitors (e.g., baloxavir-resistant PA-I38T), alongside the selection of HBV core protein mutants (such as P25G and T33N) that reduce the potency of traditional capsid modulators like GLS4, collectively highlight the urgent need for innovative strategies to overcome viral adaptability.

At the molecular level, viral resistance typically arises through five interconnected mechanisms: steric hindrance caused by amino acid substitutions that physically block drug binding, dynamic conformational remodeling of target proteins that reduces binding affinity, and alterations in protein oligomerization states that disrupt drug-mediated interactions, enhanced drug efflux through upregulated transporters, and viral genome replication errors that accelerate the selection of resistant variants. These mechanisms are not mutually exclusive; rather, they often act in concert to create robust resistance barriers. Overcoming such barriers requires a deep understanding of virus-drug interactions at the atomic scale, coupled with rational drug design strategies that anticipate and mitigate mutational escape.

This perspective synthesizes five landmark studies—focused on SARS-CoV-2, HIV-1, MPXV, IAV and HBV—to dissect the molecular basis of viral resistance and explore cutting-edge approaches in anti-resistance drug design (Fig. 1). By adopting a structure-mechanism-design framework, we aim to highlight common themes across diverse viral systems and outline translational strategies for developing durable antiviral therapies.

**Fig. 1:**
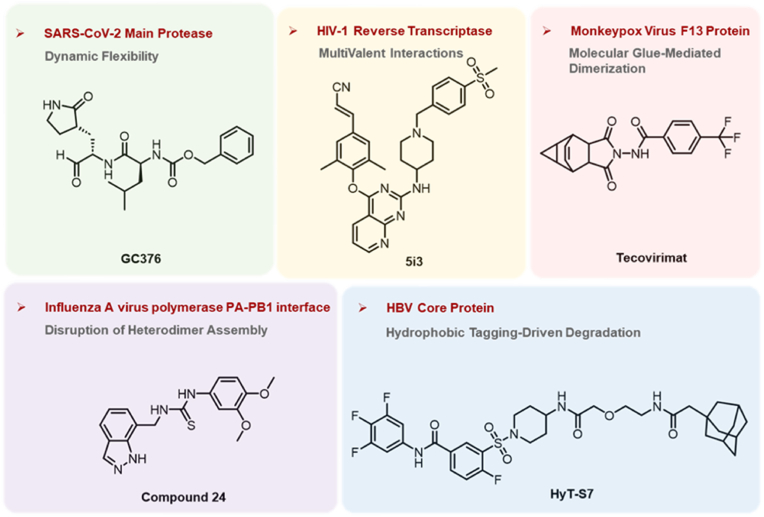
Anti-resistance drug design strategies and corresponding inhibitors for SARS-CoV-2, HIV-1, MPXV, IAV and HBV.

In a seminal study published in Science Advances [9], Neilsen et al. unraveled the resistance mechanism of SARS-CoV-2's main protease (nsp5) to the oral antiviral NIR, the active component of Paxlovid®. The E166V mutation, identified in immunocompromised patients, replaces a hydrophilic glutamate with a hydrophobic valine at position 166, introducing a β-branched side chain that creates severe steric clashes with NIR's bulky tert-butyl group at the P3 position. Crystallographic analysis of the E166V-nsp5 mutant revealed that NIR adopts two distinct conformations in the active site: in the dominant state (70% occupancy), the cyanopyrrolidine “warhead” of NIR is positioned 3.3 ​Å away from the catalytic cysteine (Cys145), too distant to form a covalent bond. Biophysical assays, including bio-layer interferometry (BLI), confirmed a dramatic reduction in binding affinity, with the dissociation constant (Kd) increasing from <1.26 ​nM for the wild-type (WT) to 3370 ​nM for the mutant—a 2670-fold decrease in potency. While the E166V mutation confers strong resistance to NIR, it incurs significant fitness costs, reducing wild-type (WA1) replicative capacity by 95% (explaining its rarity at 0.00029% of 2025 GISAID sequences). However, However, Omicron BA.1's compensatory P132H mutation halves this penalty, enabling more frequent E166V emergence—highlighting genetic background's role in resistance trajectories. The study's breakthrough lies in deciphering how the veterinary protease inhibitor GC376 circumvents E166V-mediated resistance. Unlike NIR, GC376 features a flexible benzyl ester group at the P3 position, which can adopt two major conformations (IIa and IIb) in the mutant active site. Both conformations successfully avoid steric clashes with Val166 while preserving the essential covalent bond with Cys145. These binding modes were rigorously confirmed through X-ray crystallography and molecular dynamics (MD) simulations. Notably, the benzyl group demonstrates significant rotational freedom, with a root-mean-square fluctuation (RMSF) of 2.6 ​Å. This dynamic flexibility, which we term “strategic flexibility,” enables the inhibitor to adapt within the constrained binding pocket through what might be described as a molecular “wiggle and jiggle” mechanism. The findings establish dynamic flexibility as a core principle for anti-resistance design principle, with strategies including: avoiding rigid bulky groups near mutation-prone sites, incorporating rotatable bonds/flexible linkers for conformational adjustment, and retaining reactive warheads for covalent engagement despite steric challenges. HIV-1's high mutation rate has spawned strains resistant to antiretrovirals, particularly NNRTIs [10]. The triple mutant GH9 (K101P/K103N/V108I) shows >162-fold resistance to rilpivirine (RPV) and 18-fold resistance to etravirine (ETR) by disrupting the NNRTI binding pocket (NNIBP) through altered hydrogen bond networks, π-π stacking, and geometry. Wang et al. (Science Advances) addressed this via structure-guided drug design [11], modifying lead compound 5i3 with a quinazoline core (replacing thiophenopyrimidine), enhancing interactions with conserved NNIBP aromatics (F227, W229, Y188). A C-4 cyanovinyl moiety position enables hydrophobic interactions, while C-6 sulfonylmethylpiperidine optimizes hydrogen bonding with K101/P101. Cryo-EM revealed 5i3induces a “roof-raising” effect, shifting the primer grip domain to expand the NNIBP for mutant accommodation. Its eight rotatable bonds enable dynamic adjustment, maintaining interactions despite V108I/K101P. Notably, 5i3 retains nanomolar activity against GH9 (EC_50_ ​= ​11.1 ​nM), outperforming RPV (154-fold) and ETR (9.4-fold). Resistance selection required 39 passages (vs. 13 for RPV), with unique mutations (A98G, V106I, Y181C) showing no cross-resistance to RPV or nucleoside RT inhibitors (NRTIs). It exhibits favorable pharmacokinetics (59.9% oral bioavailability in rats, minimal CYP450 inhibition), positioning it as a promising candidate for combination therapies. This work establishes key NNRTI design principles: conformational adaptability via flexible elements, and multivalent interactions (π-π stacking, hydrogen bonding, and hydrophobic effects) to resist mutational disruption. The success of 5i3 provides a validated blueprint for developing antivirals capable of overcoming existing and emerging resistant HIV-1 strains.

Tecovirimat (TPV), the first-line treatment for monkeypox, targets viral phospholipase F13 to block virion envelope formation, but resistance mutations (e.g., A295E, ΔN267) in F13 have emerged. Vernuccio et al. (Nature Microbiology) solved the 2.6 ​Å crystal structure of F13-TPV complex, revealing TPV acts as a molecular glue by binding to a 290 ​Å^3^ cavity at the F13 homodimer interface [12]. It stabilizes the dimer via hydrogen bonds (Y258-S292, N267-T291) and hydrophobic interactions (I262-I266, Y285-L289), shifting the equilibrium to 80% dimers, as measured by mass photometry. This dimerization prevents F13 from accessing its membrane-bound active conformation, thereby blocking viral envelope assembly. Resistance mutations cluster at the dimer interface: A295E introduces a negatively charged glutamate, disrupting Y285-N300 bonding and reducing interface surface area from 939 to 882 ​Å^2^. Crystallography of the A295E mutant showed partial TPV binding but weakened dimer stability, correlating with reduced antiviral efficacy. Similarly, the ΔN267 delete a key hydrogen bond donor, abolishing TPV-induced dimerization entirely. These structural observations explain clinical resistance patterns while suggesting new therapeutic strategies. The conservation F13 dimer interface across orthopoxvirines offers a broad-spectrum target. Rational design of “dimer lock” compounds that enforce oligomerization through alternative interaction networks could bypass existing resistance mechanisms, while high-throughput sequencing of the dimer interface region would enable proactive surveillance of emerging resistance variants. This work fundamentally redefines our understanding of TPV's mechanism while providing a structural roadmap for next-generation poxvirus therapeutics.

IAV's high mutation rate has caused resistance to neuraminidase inhibitors (oseltamivir H275Y) and the PA endonuclease inhibitor baloxavir (PA-I38T), necessitating alternative targets within the replication complex. Tang et al. (Acta Pharmaceutica Sinica B) targeted the PA-PB1 heterodimer interface [13]—a conserved hydrophobic groove essential for RNA-dependent RNA polymerase (RdRp) assembly—with indazole-containing compounds. Lead compound 24 (N-[(1H-indazol-7-yl)methyl]-3-(3,4-dimethoxyphenyl) acrylamide) exhibits sub-micromolar potency across multiple strains: EC_50_ 690 ​nM against A/WSN/33(H1N1), 540 ​nM against A/HK/1/68(H3N2), 230 ​nM against B/Florida/04/2006, while maintaining CC_50_ ​> ​100 ​μM in MDCK cells (selectivity index >145). Mechanistic studies confirmed that 24 binds PA-C (Kd ​= ​1.88 ​μM) and disrupts PA-PB1 interaction by 46.5% (H1) and 35.5% (H5). Cryo-EM-guided docking and MD simulations MD revealed key interactions: π–π stacking with W706, cation-π interactions with K643, and hydrogen bonding to E623—conserved across influenza types. Thermal shift assays validated K643 as a critical anchor: the K643A mutant abolished ligand-induced ΔT_m_ (+0.6 ​°C vs +17.5 ​°C for WT). In vivo, 800 ​mg/kg/day of 24 orally reduced lung viral titers to 33% of untreated controls in A/PR/8/34(H1N1)-infected mice (day 4 p.i.), comparable to 65 ​mg/kg oseltamivir acid. Though resistance studies are pending, 24 provides a scaffold for pan-influenza inhibitors, leveraging multivalent hydrophobic and electrostatic interactions against mutable viral interfaces.

Chronic hepatitis B remains incurable owing to the persistence of covalently closed circular DNA (cccDNA) and rapid selection of core protein (HBc) dimer interface. The phase-III capsid assembly modulator GLS4, for example, loses potency against P25G and T33N mutants that exhibit >66-fold shifts in EC_50_. Xu et al. (Acta Pharmaceutica Sinica B) applied hydrophobic tagging (HyT) technology to clinical sulfamoylbenzamide NVR 3–778 (EC_50_ ​= ​0.26 ​μM in HepAD38 ​cells) [14], they installed a PEG-adamantane HyT vector, yielding lead degrader HyT-S7. Cryo-EM-guided docking and MD simulations showed the adamantyl moiety inserts between HBc helices α3 and α5, mimicking a misfolded state that is preferentially routed to the autophagy–lysosome pathway. Consequently, HyT-S7 achievesr HBc degradation (DC_50_ ​= ​3.02 ​± ​0.54 ​μM) with antiviral activity (EC_50_ ​= ​0.46 ​μM, SI ​= ​62.7). Cellular thermal shift assays (ΔT_m_ ​= ​5.4 ​°C) and SPR (Kd ​= ​0.78 ​μM) confirmed direct, high-affinity engagement. Critically, HyT-S7 retained nanomolar degradation potency against a panel of 11 clinically mutants (including P25G and T33N), whereas GLS4 activity was abolished. Resistance required 25 passages under HyT-S7 pressure, twice that of GLS4, and the emergent substitutions (e.g., A119T) map outside the canonical dimer pocket, underscoring a higher genetic barrier. Pharmacokinetic evaluation in rats demonstrated 45% oral bioavailability and minimal CYP3A4 inhibition (IC_50_ ​> ​50 ​μM), supporting once-daily dosing. These findings establish HyT-mediated HBc degradation as a first-in-class paradigm that combines event-driven pharmacology with conformational plasticity, offering a blueprint for broad-spectrum antivirals resilient to core-protein resistance.

Artificial intelligence (AI) tools have revolutionized anti-resistance drug design by enabling precise prediction, efficient generation, and high-throughput optimization of drug candidates. Beyond AlphaFold2 and RoseTTAFold for mutant protein structure prediction, a suite of specialized AI tools addresses key challenges in resistance mitigation, from binding pose prediction to de novo molecule generation (Table 1). These tools synergize to streamline the design of “mutation-resilient” antivirals by integrating structural insights with dynamic adaptability.

**Table 1:** Comparison of AI tools for anti-resistance antiviral drug design.

AI Tool	Core Function	Application in Anti-Resistance Design	Key Advantages	Limitations	References
DiffDock	Binding pose prediction	Predicting drug binding to mutated targets	Accounts for ligand/protein flexibility	High computational cost for large libraries	[15]
REINVENT	Generative chemistry	De novo design of flexible/multivalent antivirals	Optimizes for resistance resilience	Generated molecules may require experimental validation of ADMET properties	[16]
MolGPT	Generative chemistry	Generating ligand libraries with targeted flexibility	Captures chemical grammar for drug-like molecules	Limited focus on target-specific interactions	[17]
AlphaFold2	Protein structure prediction	Modeling mutant proteins	High accuracy for monomeric and oligomeric structures	Limited accuracy for highly dynamic or poorly conserved regions	[18]
RoseTTAFold	Protein structure prediction	Predicting multi-chain complexes	Superior for oligomeric interfaces	Slower inference compared to AlphaFold2	[19]
DeepScreen	Virtual screening	Prioritizing hits active against resistant mutants	Integrates SAR from resistant variants	Relies on large, high-quality bioactivity datasets	[20]

Binding pose prediction tools like DiffDock [15] leverage geometric deep learning to predict drug-target interactions even for mutated proteins with altered binding pockets. Unlike traditional docking methods, DiffDock accounts for both ligand and protein flexibility, critical for modeling interactions with mutant targets, where rigid docking often fails to capture viable binding modes. Generative chemistry platforms such as REINVENT [16] and MolGPT [17] use reinforcement learning to generate novel molecules tailored to resist mutational escape. By optimizing for multi-valent interactions or dynamic flexibility these tools design compounds that maintain affinity across diverse mutant backgrounds. For example, REINVENT has been used to generate NNRTI analogs with enhanced adaptability to RT mutations by prioritizing scaffolds with extended π-systems and hydrogen-bonding moieties. Structure prediction tools like AlphaFold2 [18] and RoseTTAFold [19] remain foundational, providing high-confidence models of mutant proteins when experimental structures are unavailable. These models serve as inputs for downstream docking and MD simulations, enabling in silico validation of anti-resistance candidates before experimental testing. Virtual screening tools such as DeepScreen [20] integrate deep learning with bioactivity data to prioritize compounds likely to retain efficacy against resistant mutants. By learning from structure-activity relationships (SAR) of existing antivirals and their resistant variants, DeepScreen accelerates hit identification, reducing the need for costly high-throughput screening.

Pharmacokinetic Optimization: Compounds like 5i3 require extended half-life optimization for once-daily dosing, potentially through structural modifications or novel formulations (e.g., long-acting injectables).

Broad-Spectrum Efficacy: While targeting conserved domains (e.g., nsp5's Cys145, F13's dimer interface) enhances pan-viral activity, species-specific differences (e.g., Clade I MPXV's reduced responsiveness to TPV) necessitate mechanistic clarification. This is particularly critical for influenza, where antigenic drift complicates cross-strain efficacy, and for HBV, where genotypic variations (e.g., genotype C vs. B) affect drug responsiveness.

Pro-Drug Design: Activating drugs in specific cellular compartments (e.g., viral replication factories) could enhance local concentration and reduce systemic toxicity.

Combination Therapies: Pairing anti-resistance compounds with drugs targeting orthogonal pathways (e.g., NNRTIs ​+ ​NRTIs for HIV-1, TPV ​+ ​DNA polymerase inhibitors for MPXV) may delay resistance emergence through synergistic pressure. For influenza, combining neuraminidase inhibitors with cap-dependent endonuclease inhibitors has shown promise, while for HBV, nucleos(t)ide analogs paired with capsid modulators offer a multi-pronged approach.

CRISPR-Based Resistance Prevention: Editing viral target genes to introduce “self-limiting” mutations (e.g., essential residues intolerant to drug resistance mutations) could preempt adaptive evolution.

The structural and mechanistic paradigms revealed across SARS-CoV-2, HIV-1, MPXV, IAV, and HBV collectively underscore the potential for biomarker-driven clinical strategies that bridge atomic-scale resistance mechanisms with therapeutic decision-making. For SARS-CoV-2, the dynamic flexibility of GC376 in overcoming E166V-mediated steric hindrance suggests that pre-treatment assessment of nsp5 dimer stability and mutation profiling (e.g., P132H compensatory mutations) could stratify patients for protease inhibitor regimens, while real-time crystallography of clinical isolates might monitor conformational adaptation during therapy. Similarly, HIV-1's NNRTI resistance landscape demands conformational monitoring of the RT “roof-raising” effect induced by 5i3, where cryo-EM characterization of patient-derived quaternary structures could guide combination therapies with NRTIs to suppress emergent variants like A98G/V106I. The molecular glue mechanism of MPXV's tecovirimat highlights dimer: monomer equilibrium as a pharmacodynamic biomarker, measurable through mass photometry of lesion-derived F13 proteins, particularly in immunocompromised hosts harboring interface mutations (A295E/ΔN267). Influenza's conserved PA-PB1 interface offers opportunities for pan-viral indazole-based inhibitors, where deep mutational scanning of W706/K643 conservation could prioritize compound 24 derivatives for patients with PA-I38T or oseltamivir resistance, complemented by co-immunoprecipitation assays quantifying polymerase complex disruption. For HBV, the resilience of HyT-S7 against core protein mutants (P25G/T33N) positions HBc aggregation state—visualized via liver biopsy cryo-EM—as a critical determinant for degrader efficacy, with autophagy flux markers (LC3B-II/p62) tracking on-target activity. These pathogen-specific approaches converge on a unified clinical vision: adaptive platform trials targeting shared vulnerabilities (e.g., oligomerization interfaces across nsp5, F13, and PA-PB1) with modular arms that incorporate real-time genomic surveillance (GISAID, GenBank) and structure-guided companion diagnostics (CRISPR-based mutation detection) to dynamically adjust therapeutic combinations against evolving resistance. Such integration of high-resolution structural biology with precision medicine frameworks promises to transform antiviral therapy from reactive to preemptive—a paradigm shift essential for outmaneuvering viral adaptability.

The widespread use of antiviral drugs demands careful stewardship to minimize resistance selection. Critical priorities include implementing real-time genomic surveillance systems to track emerging resistance mutations across regions, with focused monitoring of high-risk variants such as SARS-CoV-2 E166V and HIV-1 K103N. Equally important is developing ethical frameworks to ensure equitable global access to next-generation antivirals, particularly in resource-limited settings where resistance surveillance remains inadequate. Establishing evidence-based guidelines for therapeutic drug monitoring (TDM) that account for host-pathogen pharmacodynamic interactions and promoting antimicrobial stewardship programs integrating resistance profiling with precision dosing strategies are also essential. These measures should be supported by international collaboration to standardize resistance reporting and optimize treatment algorithms based on local epidemiological patterns.

The studies discussed herein exemplify the power of integrating structural biology, MD, and medicinal chemistry to decode viral resistance and engineer resilient therapies. From GC376's dynamic flexibility to 5i3's conformational adaptability and TPV's molecular glue mechanism, each highlights a unique strategy to counteract steric, dynamic, and oligomerization-based resistance.

Collectively, these findings underscore that viral resistance is not an insurmountable barrier but a solvable problem rooted in molecular mechanics. As we enter an era of predictive anti-resistance design—powered by AI, high-resolution structural techniques, and systems biology—the goal of developing “evergreen” antivirals capable of anticipating and neutralizing mutational escape becomes increasingly attainable. By embracing these multidisciplinary approaches [21], we can transform the current reactive paradigm of antiviral development into a proactive, resilient strategy, ensuring lasting protection against present and future viral threats.

## CRediT authorship contribution statement

**Mei Wang:** Writing – original draft, Conceptualization. **Haiyong Jia:** Writing – review & editing, Visualization. **Xinyong Liu:** Writing – review & editing, Visualization. **Peng Zhan:** Writing – review & editing, Visualization.

## Ethics approval

Not applicable.

## Declaration of generative AI in scientific writing

Not applicable.

## Funding information

The authors are supported by the Key Research and Development Program, Ministry of Science and Technology of the People's Republic of China (No. 2023YFC2606500,2023YFE0206500).

## Declaration of competing interests

The authors declare that they have no known competing financial interests or personal relationships that could have appeared to influence the work reported in this paper.

## Data Availability

Not applicable.
